# Supplementary material for: Genome-Wide Association Study Meta-Analysis Elucidates Genetic Structure and Identifies Candidate Genes of Teat Number Traits in Pigs
Source: Int J Mol Sci. 2023 Dec 29;25(1):451. doi: 10.3390/ijms25010451 (PMC10779318; doi:10.3390/ijms25010451)
Supplement: Supplementary file 1 [file ijms-25-00451-s001.zip › Supplementary Figures.pdf]

Genome-wide association study meta-analysis elucidates genetic structure and identifies candidate genes of teat number traits in pigs

Tingting Li, Pengchong Wan, Qing Lin, Chen Wei, Kaixuan Guo, Xiaojing Li, Yujin Lu, Zhe Zhang*, Jiaqi Li*

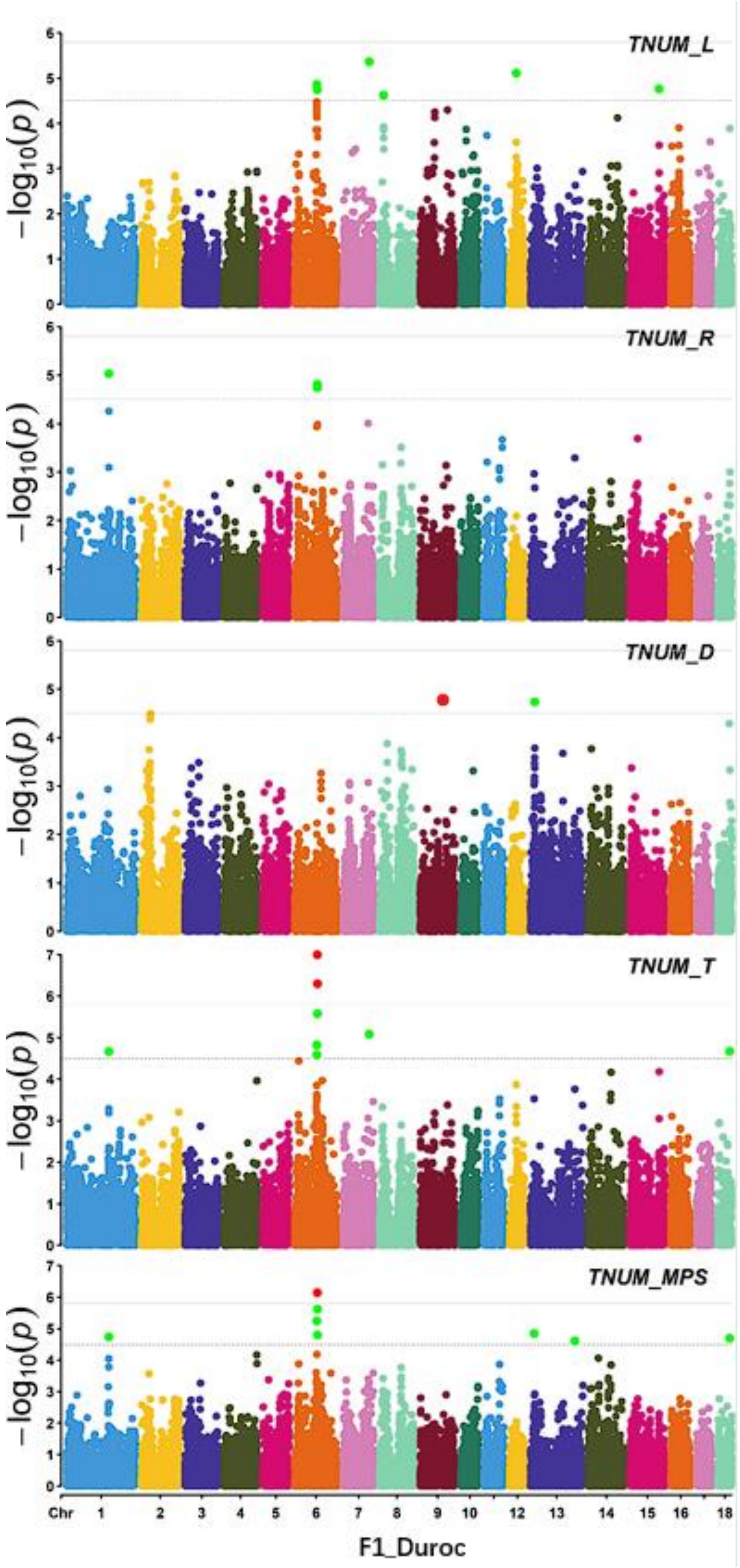
TNUM_L
TNUM_R
TNUM_D
TNUM_T
TNUM_MPS
−log10(p)
Chr 1 2 3 4 5 6 7 8 9 10 12 13 14 15 16 18
F1_Duroc

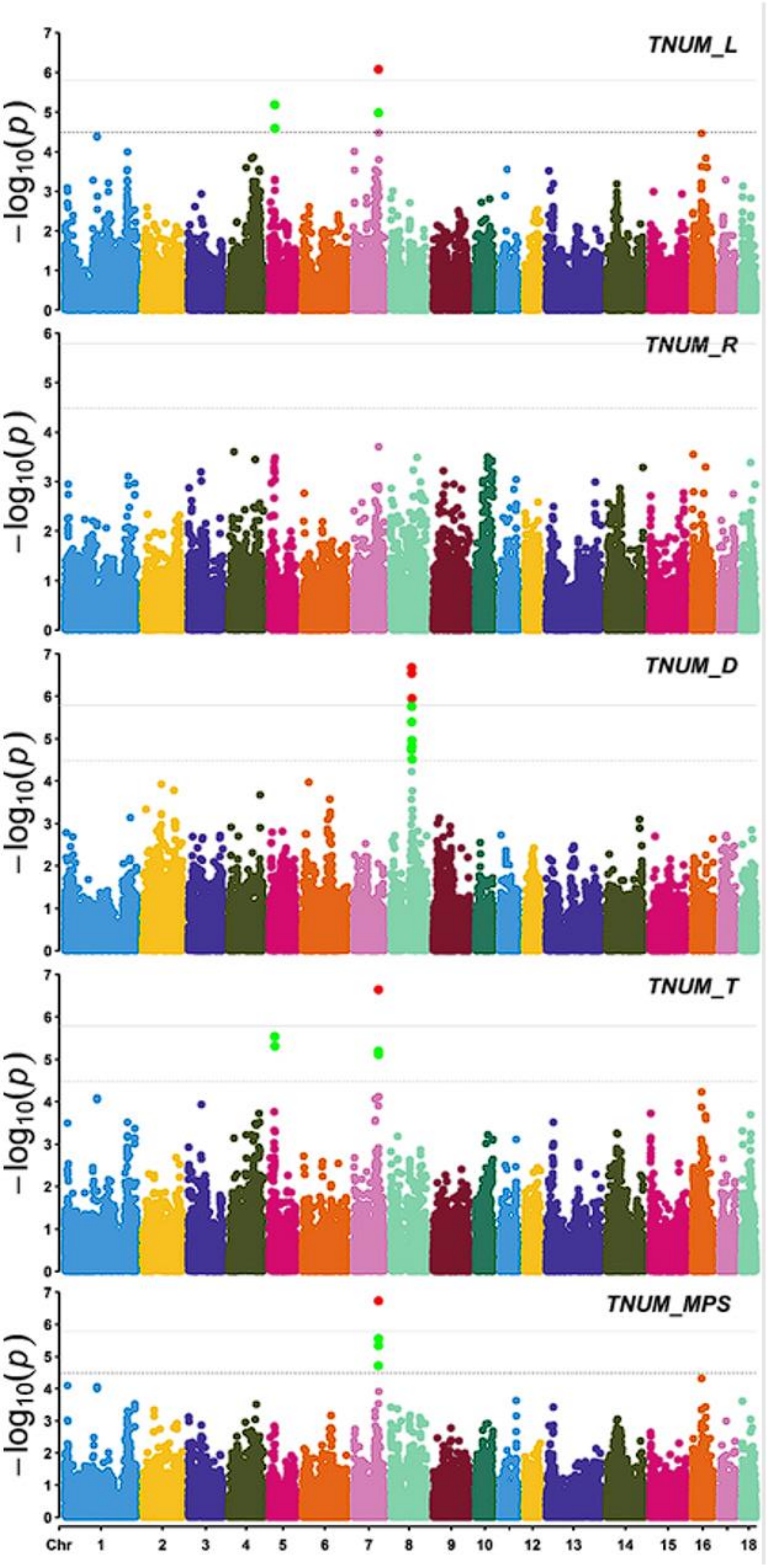
TNUM_L
TNUM_R
TNUM_D
TNUM_T
TNUM_MPS
$-\log_{10}(p)$
Chr 1 2 3 4 5 6 7 8 9 10 12 13 14 15 16 18

F1_Landrace

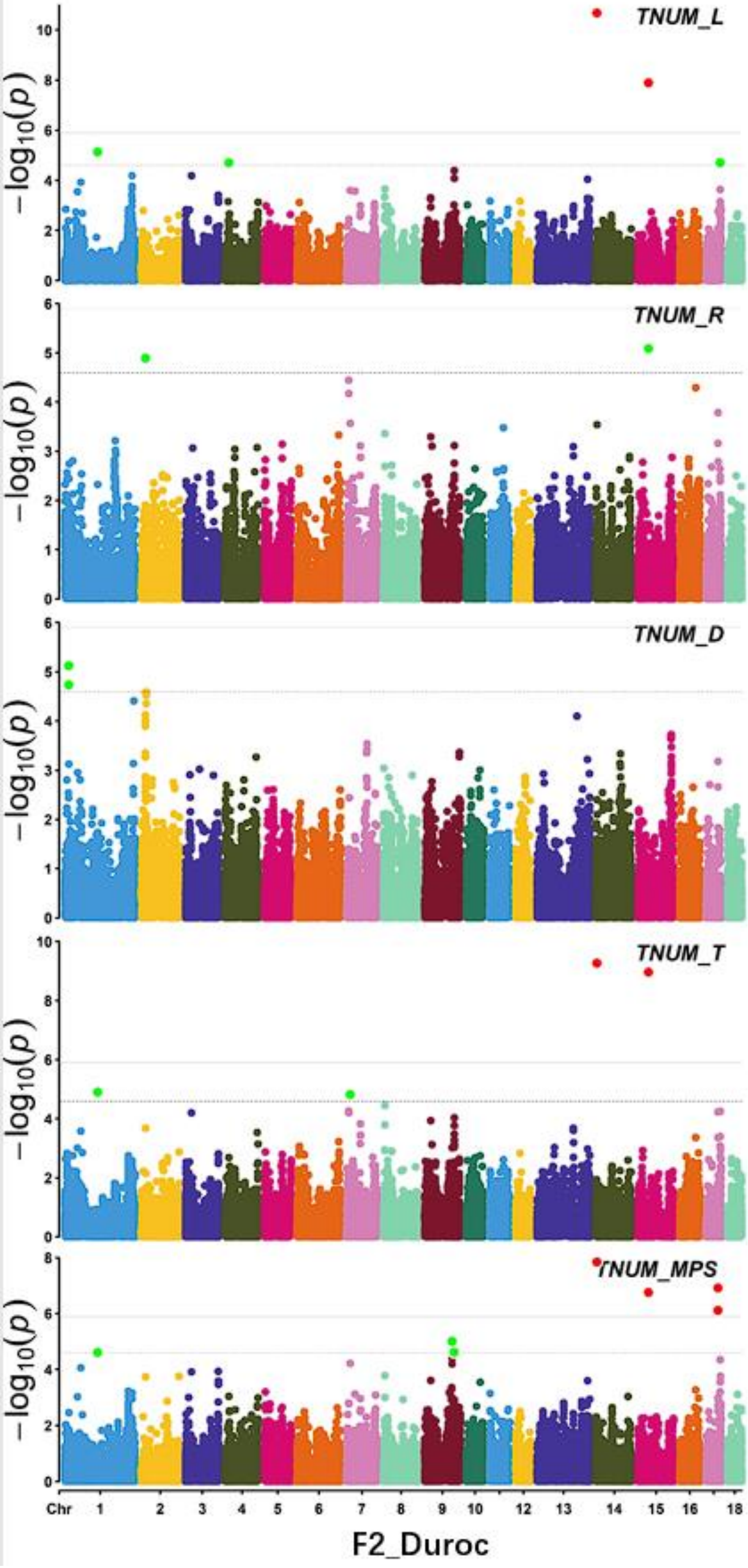
TNUM_L
TNUM_R
TNUM_D
TNUM_T
TNUM_MPS
−log10(p)
Chr
F2_Duroc

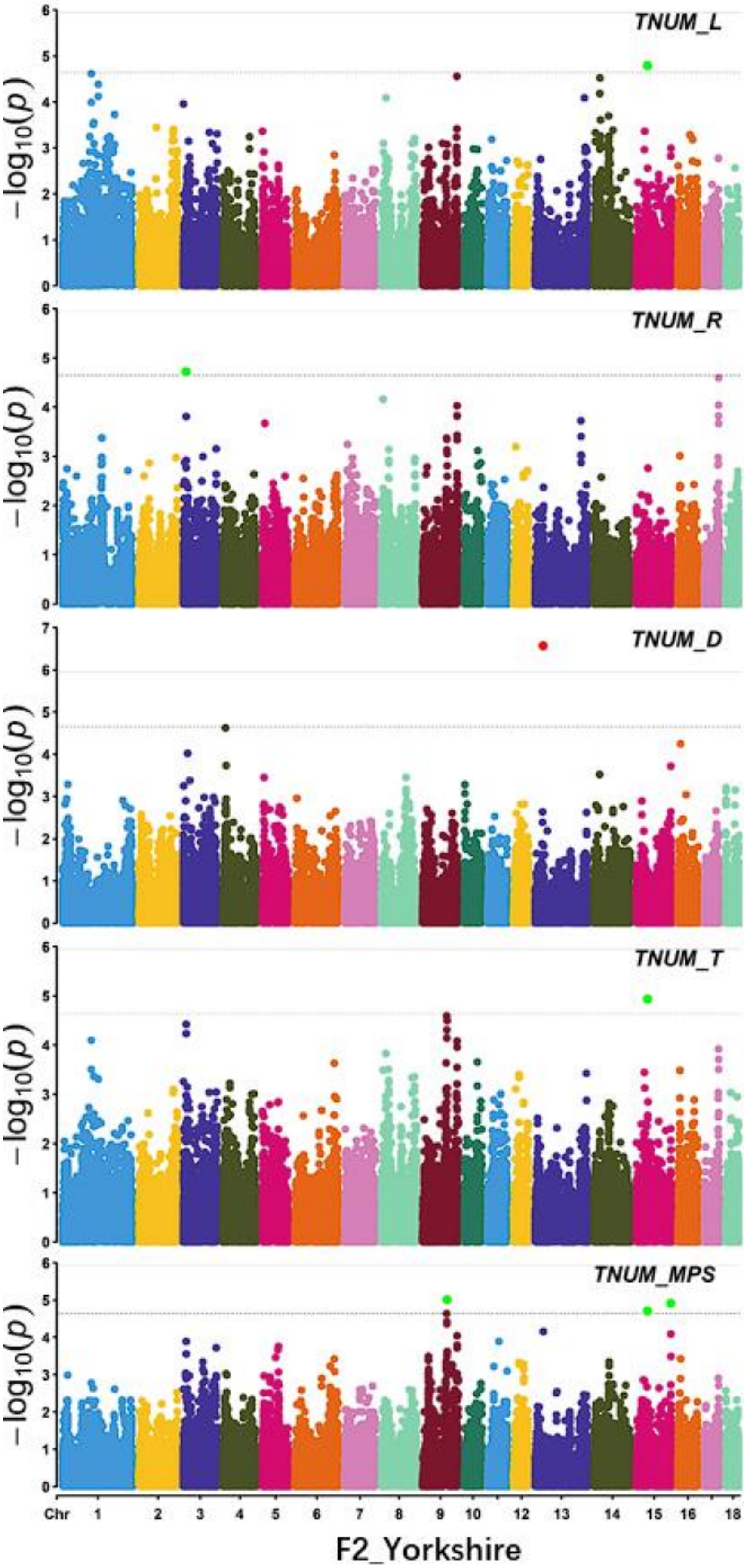

TNUM_L
TNUM_R
TNUM_D
TNUM_T
TNUM_MPS
−log10(p)
Chr 1 2 3 4 5 6 7 8 9 10 12 13 14 15 16 18
F2_Yorkshire

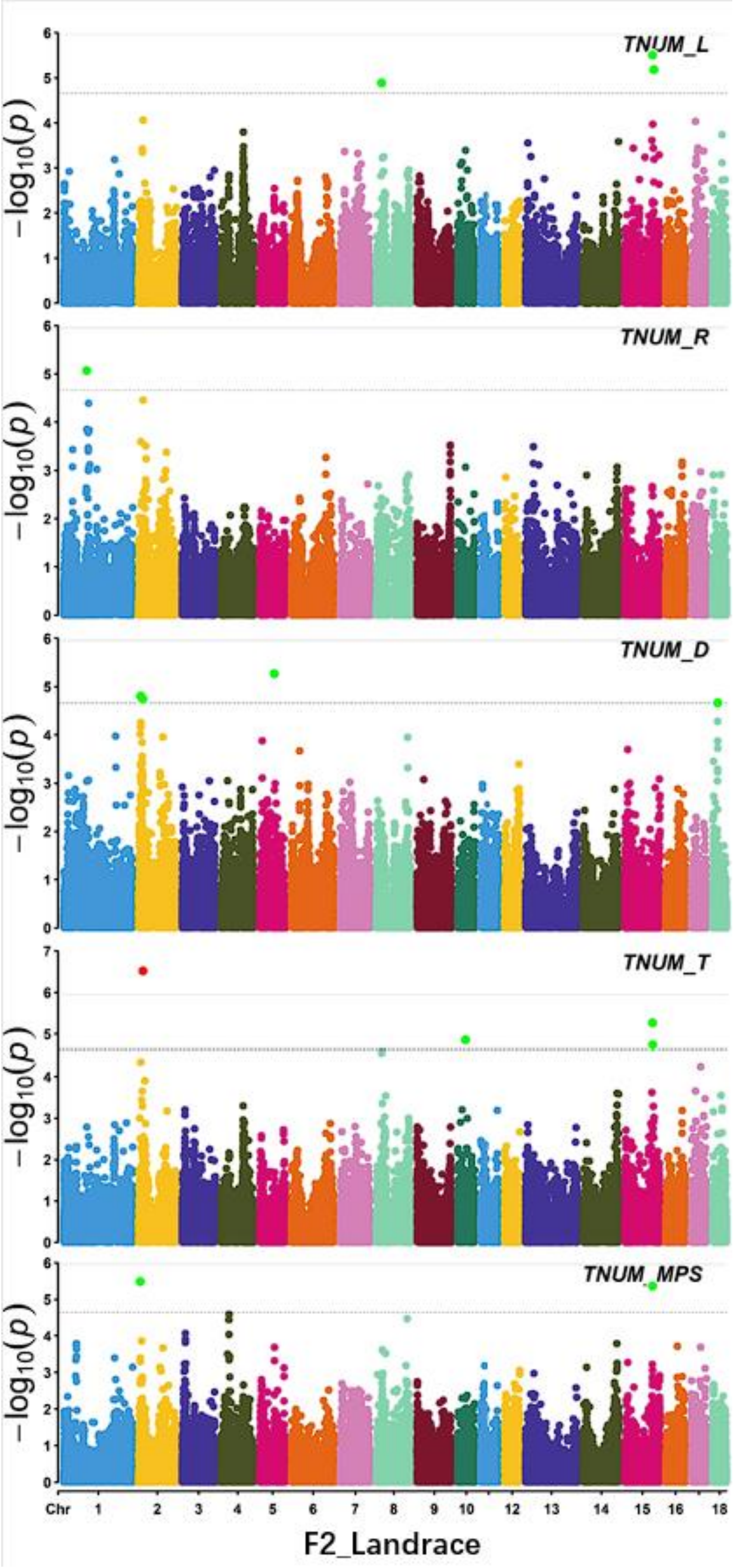

TNUM_L
TNUM_R
TNUM_D
TNUM_T
TNUM_MPS
−log10(p)
Chr 1 2 3 4 5 6 7 8 9 10 12 13 14 15 16 18
F2_Landrace

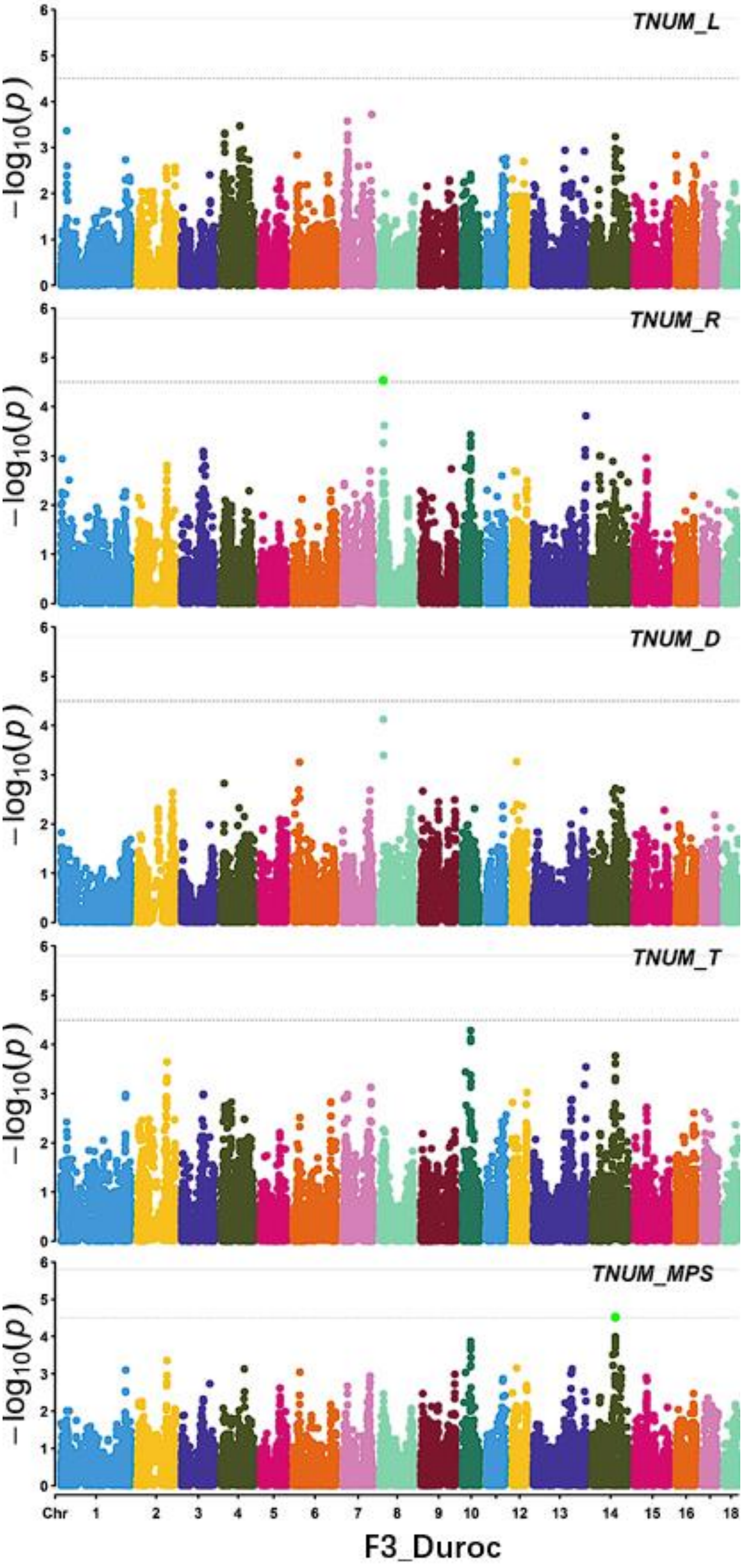

TNUM_L
TNUM_R
TNUM_D
TNUM_T
TNUM_MPS
−log10(p)
0
1
2
3
4
5
6
Chr 1 2 3 4 5 6 7 8 9 10 12 13 14 15 16 18
F3_Duroc

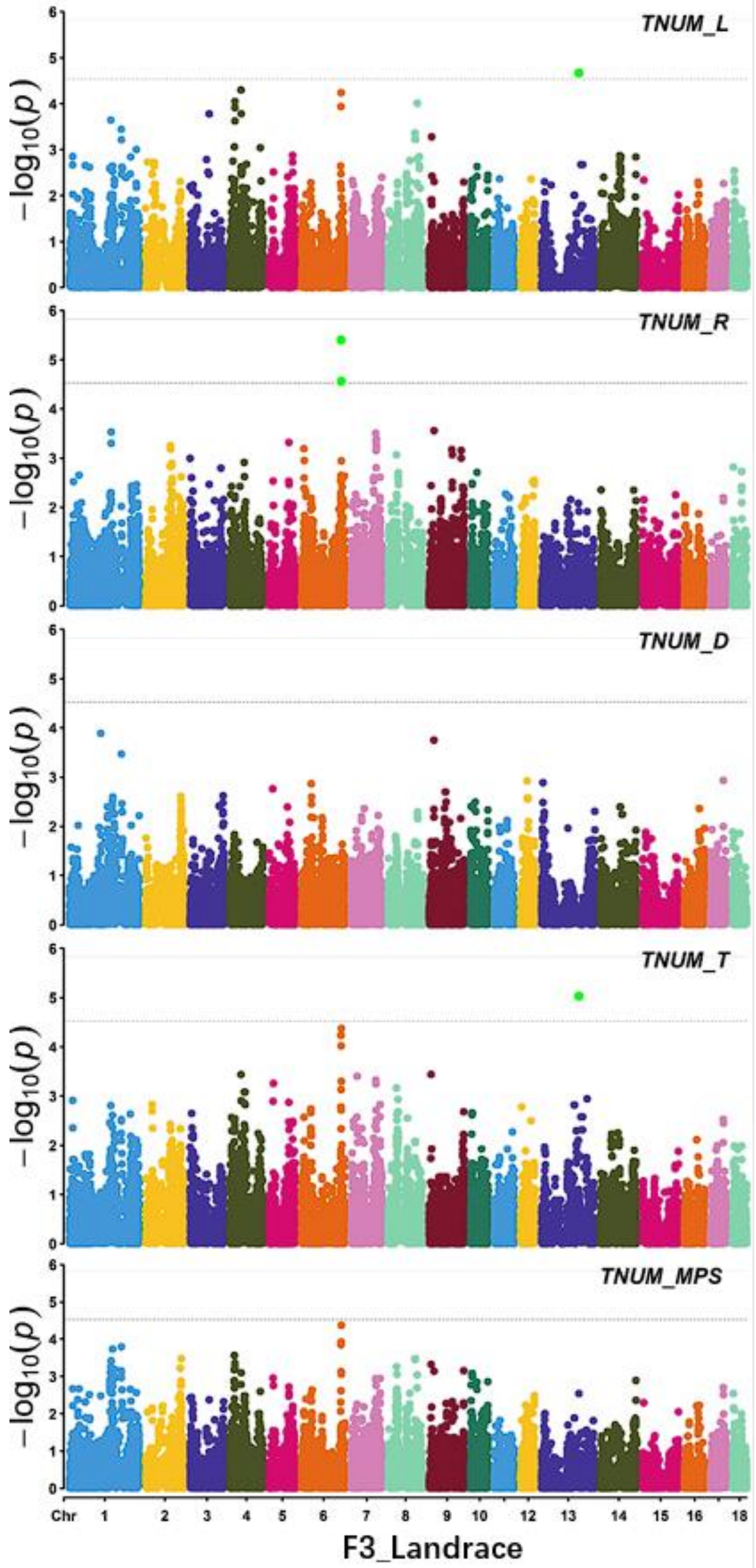
TNUM_L
TNUM_R
TNUM_D
TNUM_T
TNUM_MPS
$-\log_{10}(p)$
Chr 1 2 3 4 5 6 7 8 9 10 12 13 14 15 16 18
F3_Landrace

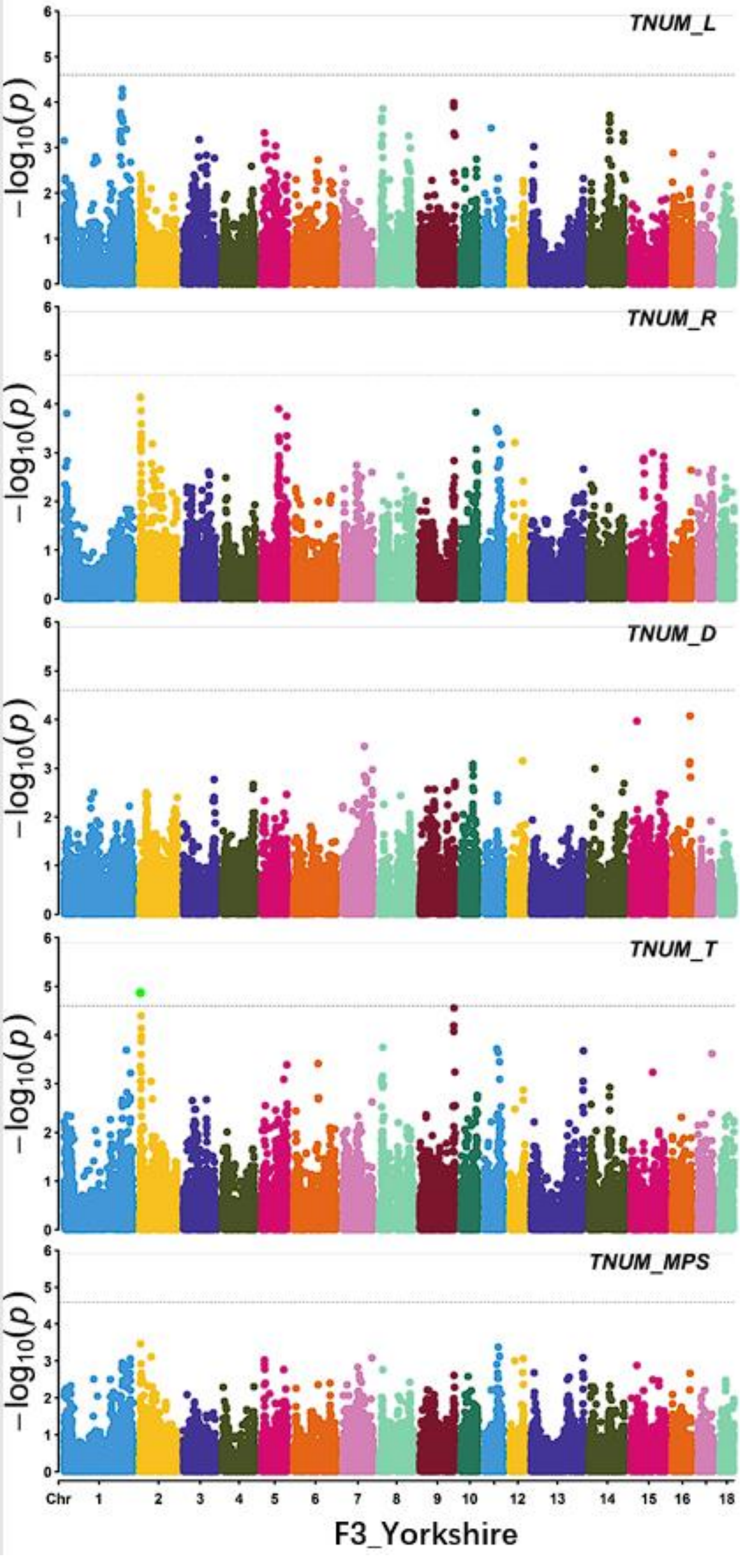

TNUM_L
TNUM_R
TNUM_D
TNUM_T
TNUM_MPS
$-\log_{10}(p)$
Chr 1 2 3 4 5 6 7 8 9 10 12 13 14 15 16 18
F3_Yorkshire

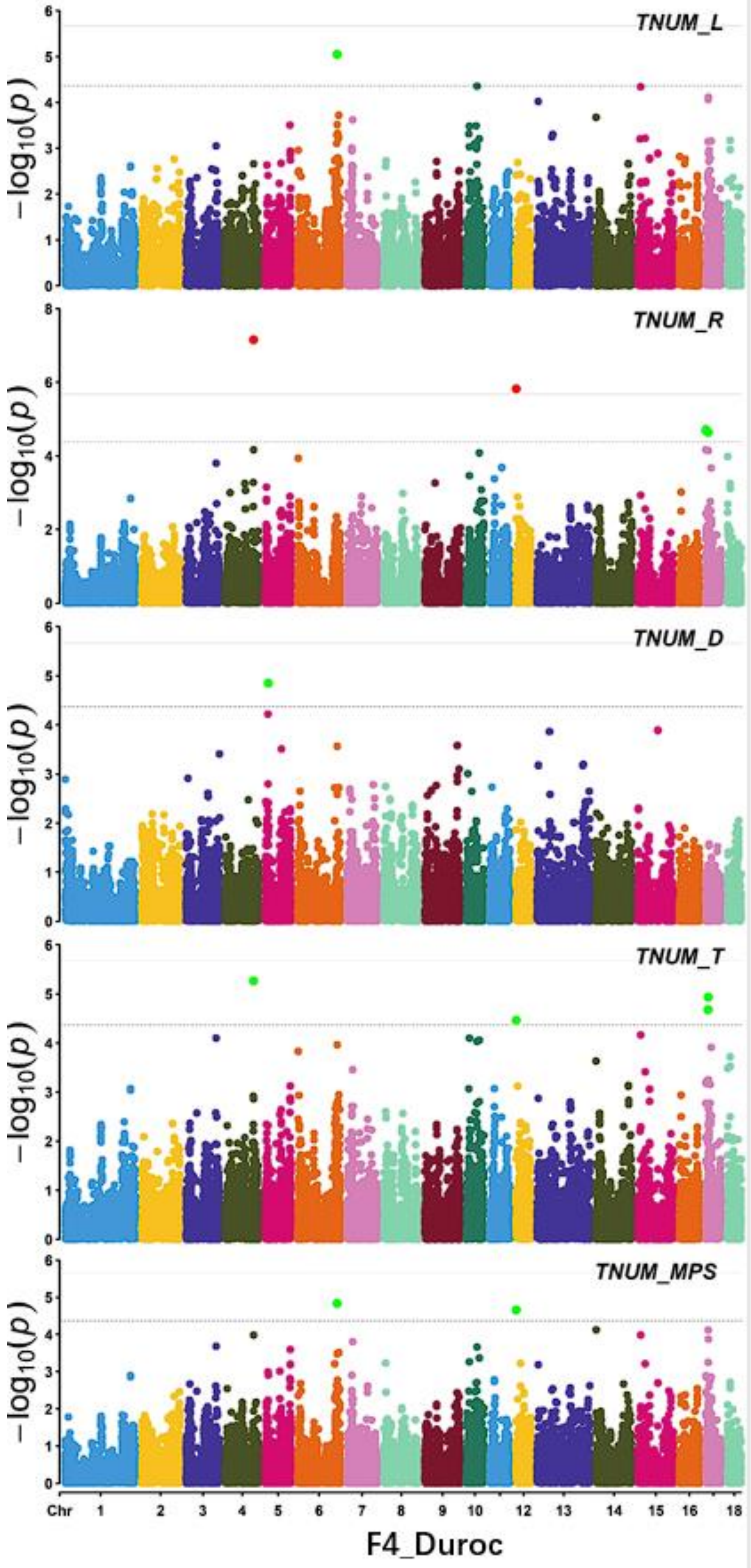
TNUM_L
TNUM_R
TNUM_D
TNUM_T
TNUM_MPS
$-\log_{10}(p)$
Chr 1 2 3 4 5 6 7 8 9 10 12 13 14 15 16 18
F4_Duroc

Figure S1 The 45 GWAS Manhattan plots show the results of a single population GWAS, with significance thresholds of 0.05/SNP and 1/SNP, respectively.

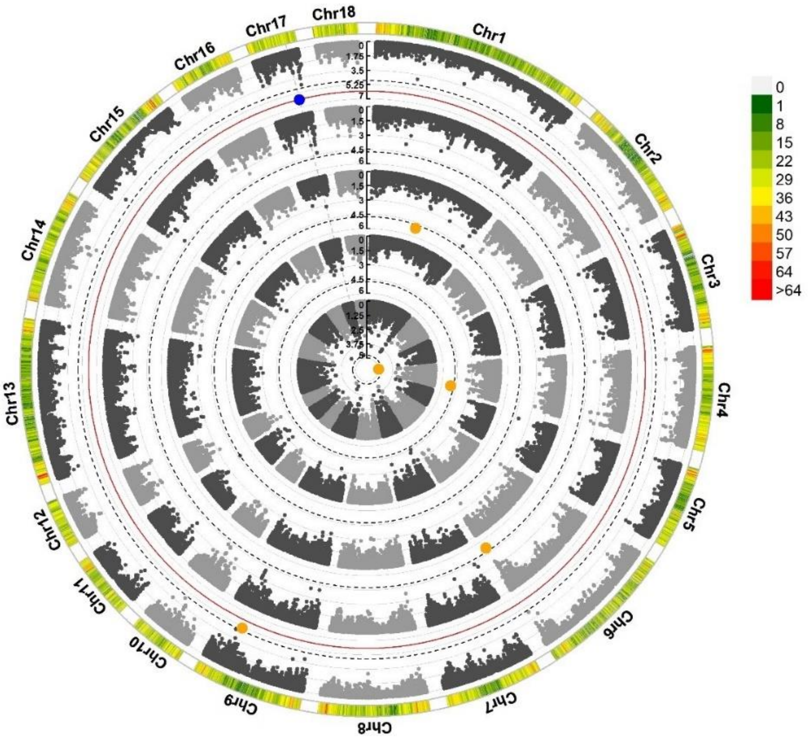

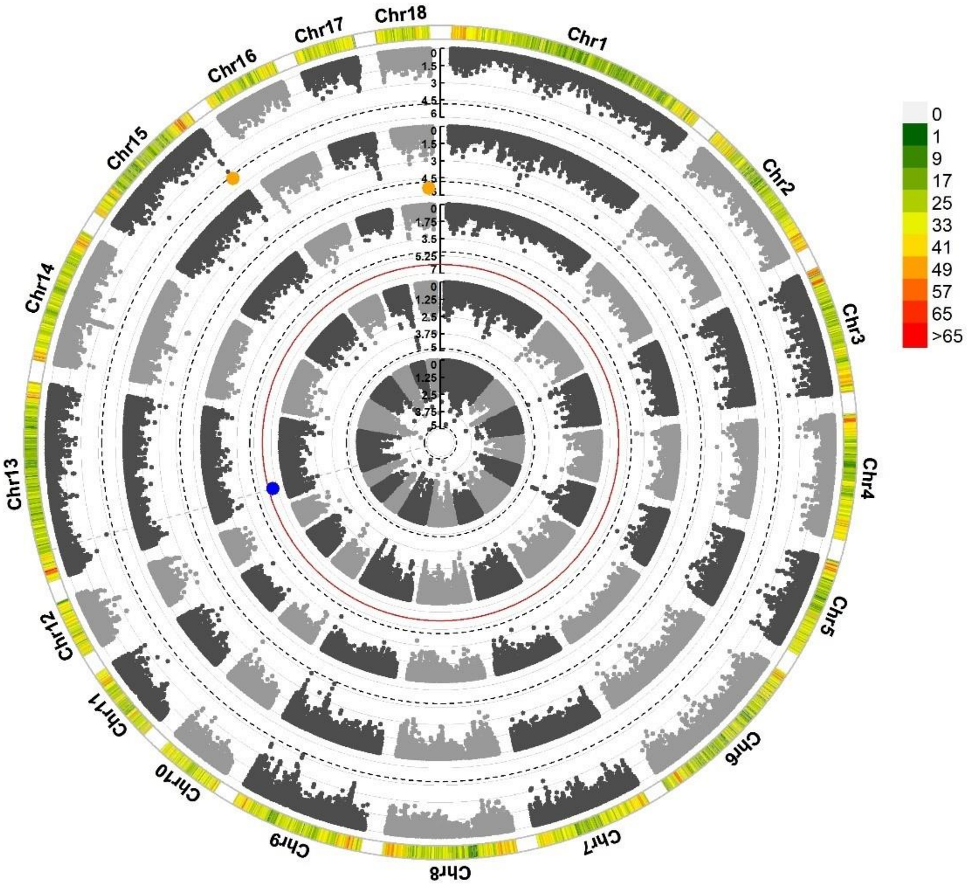

Figure S2 Circular Manhattan of meta-analysis for single breed, from left to right are Yorkshire pigs and Duroc pigs respectively. The blue dots represent SNPs whose P-values exceed the genomic significance threshold; the orange dots represent SNPs whose P values exceed the threshold of chromosomal significance.

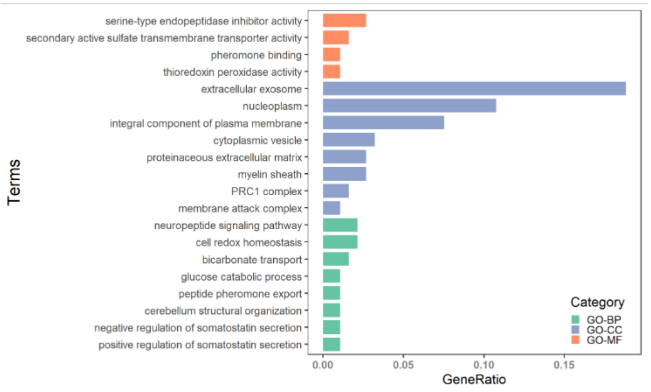

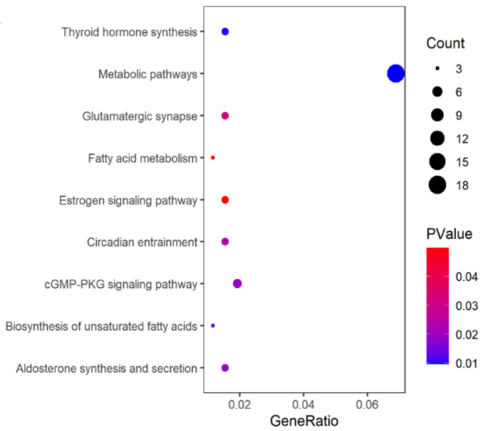

Figure S3 Annotated gene GO and KEGG enrichment results

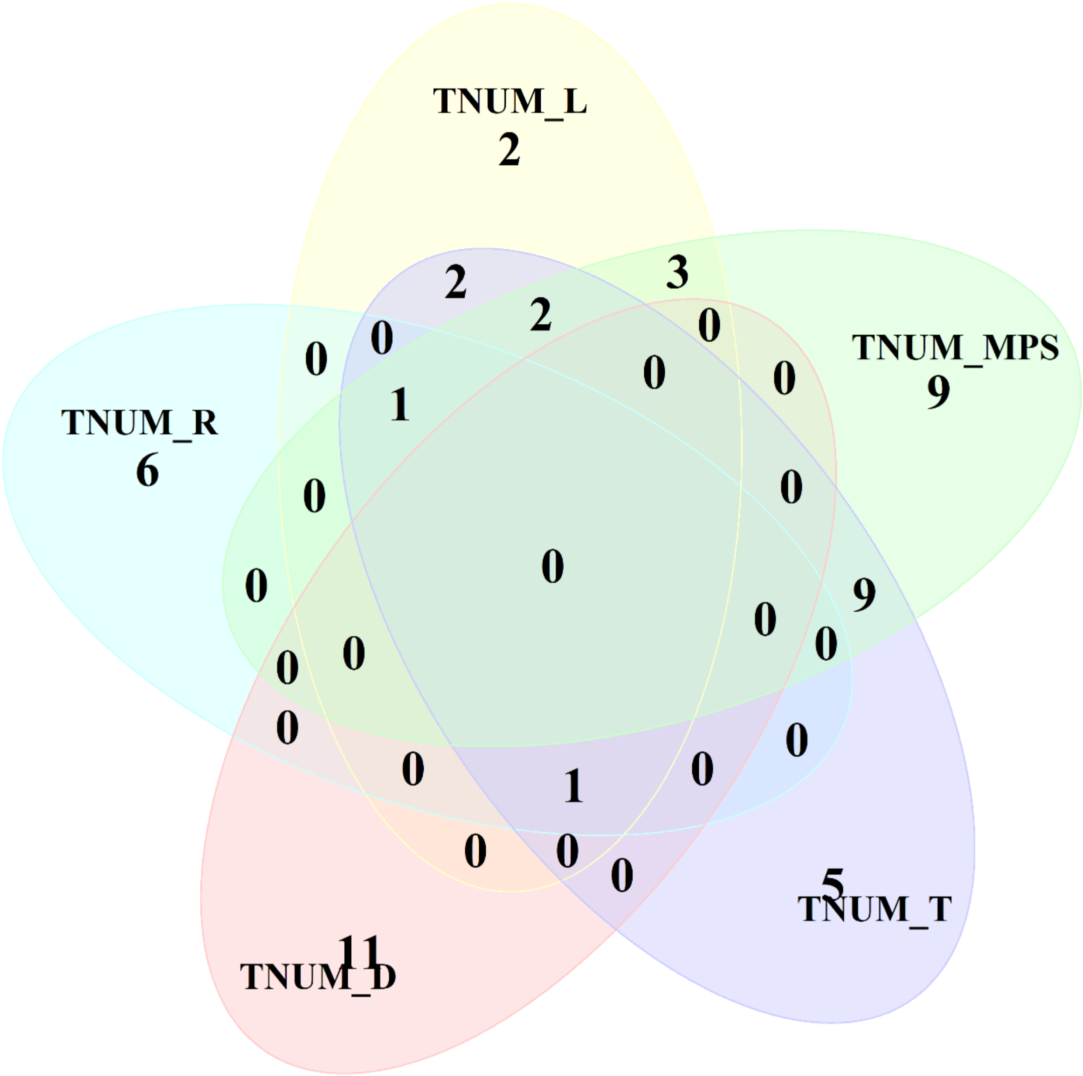

Figure S4 the Venn diagram of genes annotated with significant SNPs for each trait
